# Non-Negative Matrix Factorization Based on Smoothing and Sparse Constraints for Hyperspectral Unmixing

**DOI:** 10.3390/s22145417

**Published:** 2022-07-20

**Authors:** Xiangxiang Jia, Baofeng Guo

**Affiliations:** School of Automation, Hangzhou Dianzi University, Hangzhou 310018, China; jxxhdu@163.com

**Keywords:** hyperspectral unmixing, non-negative matrix factorization, reweighted sparseness, total variation, piecewise smoothness constraint

## Abstract

Hyperspectral unmixing (HU) is a technique for estimating a set of pure source signals (end members) and their proportions (abundances) from each pixel of the hyperspectral image. Non-negative matrix factorization (NMF) can decompose the observation matrix into the product of two non-negative matrices simultaneously and can be used in HU. Unfortunately, a limitation of many traditional NMF-based methods, i.e., the non-convexity of the objective function, may lead to a sub-optimal solution. Thus, we put forward a new unmixing method based on NMF under smoothing and sparse constraints to obtain a better solution. First, considering the sparseness of the abundance matrix, a weight sparse regularization is introduced into the NMF model to ensure the sparseness of the abundance matrix. Second, according to the similarity prior of the same feature in the adjacent pixels, a Total Variation regularization is further added to the NMF model to improve the smoothness of the abundance map. Finally, the signatures of each end member are modified smoothly in spectral space. Moreover, it is noticed that discontinuities may emerge due to the removal of noisy bands. Therefore, the spectral data are piecewise smooth in spectral space. Then, in this paper, a piecewise smoothness constraint is further applied to each column of the end-member matrix. Experiments are conducted to evaluate the effectiveness of the proposed method based on two different datasets, including a synthetic dataset and the real-life Cuprite dataset, respectively. Experimental results show that the proposed method outperforms several state-of-the-art HU methods. In the Cuprite hyperspectral dataset, the proposed method’s Spectral Angle Distance is 0.1694, compared to the TV-RSNMF method’s 0.1703, *L*_1/2_NMF method’s 0.1925, and VCA-FCLS method’s 0.1872.

## 1. Introduction

The hyperspectral sensor simultaneously acquires hundreds of continuous spectral bands in the wavelength, ranging from 0.4 to 2.5 μm. Therefore, the hyperspectral images (HSI) have a high spectral resolution, which promotes the development of quantitative remote sensing applications [1]. However, due to the widespread presence of mixed pixels resulting from the low spatial resolution of the sensor, i.e., a pixel is mixed with several end-member signatures, the accuracy of identification of the images can be affected [2]. Thus, the mixed pixels should be decomposed into a series of end members and their proportional fractions to improve the performance of HSI analysis [3]. HU is a technique to find out the pure spectra (i.e., end members) and their specific percentage (i.e., abundance) for every pixel, which has been extensively studied in recent years to solve the mixed pixel problem.

According to the previous studies, HU algorithms can be divided into two categories. The first group of methods consists of several independent steps, namely end-member estimation and abundance estimation. The end-member estimation algorithms mainly include PPI [4], N-FINDR [5], and VCA [6], which are usually based on the pure pixel assumption, i.e., it is assumed that there exists one pure pixel at least for each sort of material in HSI. However, in some cases, the assumption is not reliable. To solve this problem, some improved methods have been proposed by related researchers. For example, Miao et al. [7] proposed to find the simplex that can contain all pixels with minimum volume, and the vertices corresponding to the simplex are the desired end members. After the extraction of the end member, it is followed by the abundance estimate. The fully constrained least squares (FCLS) method [8] is the most common method of estimating abundance. Satisfactory results can be obtained using this kind of end-member estimation combined with abundance estimation, but the performance of HU is heavily influenced by the accuracy of end-member estimation.

To avoid the above problems, related researchers have proposed a secondary category of the HU method from a perspective of statistical analysis, i.e., taking HU as a blind source separation problem. Such methods mainly include independent component analysis (ICA) [9] and non-negative matrix factorization (NMF) [10], where the ICA method assumes that the end-member matrix and the abundance matrix are independent of each other. However, the sum of the fractions proportional to each material is 1, so the independence assumption of ICA cannot be guaranteed in HSI. It can only be used as an approximate solution for HU in some cases. Hyperspectral data is decomposed into the end-member matrix and abundance matrix by HU, and the observation matrix is decomposed into a product of two non-negative matrices by the NMF-based method, which better fits the needs of HU and has no need to satisfy the assumption of pure pixels. Therefore, this method is widely used for HU.

Unfortunately, the objective function of the classic NMF method is non-convex, which means the result may fall into local minima. In order to solve this problem, different constraints based on the characteristics of the HSI were introduced. For example, the distribution of end member in HSI is clustered so that most pixels are mixed by some particular end members instead of all of them. Thus, the columns of the abundance matrix are sparse. Accordingly, researchers proposed to use $L_{1}$ norm [11] to promote the sparsity of the results. However, $L_{1}$ norm cannot enforce further sparsity when the full additivity constraint of the material abundances is used. Then, Qian et al. proposed an HU method based on the $L_{1/2}$ norm [12], which can obtain more accurate results than that of $L_{1}$. However, this article only considers the sparse feature of HSI. For hyperspectral data, due to its low spatial resolution, the abundances of each end member vary smoothly in spatial space. However, discontinuities may occur due to abrupt changes in abundance. Therefore, the spectral data are piecewise smooth in the spatial domain. Hua et al. proposed an adaptive abundance smoothing (AAS) autoencoder network to promote abundance smoothing [13]. Specifically, a multilayer encoder is used to obtain the abundance, and then the input layer is constructed using a single-layer decoder. Finally, $L_{1/2}$ is applied to promote sparsity. In addition, He et al. proposed the use of Total Variation (TV) regularization to increase the piecewise smoothing of abundance [14], where TV regularization can be treated as a denoising process of the abundance map, which can improve the robustness to noise. In addition, the spectrum is smoothly varied in the wavelength space by the high spectral resolution of HSI [15]. However, discontinuities may occur owing to the removal of noisy bands in wavelength space. Therefore, the spectra data are actually piecewise smooth in the spectral domain. Thus, Sen et al. proposed a piecewise smoothness constraint for HU [16]. It is also noticed that a new variant of NMF-based algorithms was proposed in [17], which showed great potential for many image processing applications due to its sparseness constraint and natural incorporation of local information. Apart from the above studies, some new NMF-related hyperspectral unmixing methods have been put forward in recent years, such as the novel low-rank factorization-based methods [18,19,20], and the VCA-FCLS that is used as an initialization step in [18]. Future work will be carried out to explore the relationship between the novel low-rank tensor and the non-negative matrix factorization and compare their performances. Although the above methods have achieved better results, there is still extra room for improvement.

To further improve the accuracy of HU, piecewise smoothing constraints and sparsity constraints are integrated into the NMF objective function, and an improved HU model is put forward. Specifically, in this paper, the reweighted sparsity and TV norm constraint [14] are proposed to promote the sparsity and smoothness of the abundance map, followed by the end-member smoothing constraint. First, an unconstrained NMF decomposition is performed, and the spectral difference of the adjacent bands is calculated as a measure of smoothness according to the obtained result. In addition, different smoothing weights are assigned according to different smoothness to further reduce the solution space of the NMF-based method, and the optimal solution can be found more accurately. In summary, our main contribution is to extend the reweighed sparsity and the piecewise smoothing constraints in the abundance map, which were introduced in [14], to a more specific scenario, where smoothing on the end-element spectra is also required.

The rest of this paper is organized as follows: In Section 2, we first introduce the linear spectral mixing model, followed by a brief description of the NMF. The sparsity and piecewise smoothing of hyperspectral abundance maps and the piecewise smoothing of end-member spectral are discussed in Section 3. The model proposed in this paper is described at the end of this section. The numerical results for a synthetic dataset and a real dataset are reported in Section 4. Section 5 is the conclusion with suggestions for future work.

## 2. Linear Spectral Mixture Model

In the linear mixing model, the spectral signal of a pixel is mixed by a set of end-member spectral signatures linearly according to the abundance fractions. It is defined as

(1) y=As+e

where $y\in R^{B\times 1}$ denotes a *B*-dimensional spectral vector. *B* is the number of spectral bands. $A\in R^{B\times K}$ is an end-member matrix, and each column corresponds to an end-member signature. *K* is the number of the end member, $s\in R^{K\times 1}$ is the abundance vector of a pixel, and $e\in R^{B\times 1}$ represents the Gaussian noise.

The linear mixing model for mixed pixels in HSI is expressed in the following matrix form

(2) $$\begin{array}{l} Y=AS+E\\ s.t.\;1_{K}^{T}S=1_{N}^{T},\;A\geq 0,S\geq 0 \end{array}$$

where $Y=\left[y_{1},\ldots ,y_{N}\right]\in R^{B\times N}$ is the hyperspectral data with *N* pixels and *B*-bands, $S=[s_{1},\ldots ,s_{N}]\in R^{K\times N}$ is the abundance matrix, $E\in R^{B\times N}$ represents the Gaussian noise, and $1_{K}^{T}=\left[1,\ldots ,1\right]\in R^{1\times K}$ is an all-one vector with size *K*.

The observation matrix is decomposed into the product of two non-negative matrices by the NMF-based method, and the classical NMF problem is expressed as

(3) Y≈AS

The end-member spectral matrix $A\in R^{B\times K}$ and the abundance matrix $S\in R^{K\times N}$ can be solved by minimizing the difference between Y and AS. The objective function of NMF based on the Euclidean distance is defined as follows:

(4) $$\underset{A,S}{\min}\frac{1}{2}{\left\|Y-AS\right\|}_{F}^{2},s.t.\;A\geq 0,S\geq 0$$

where ${\left\|\cdot \right\|}_{F}$ is the Frobenius norm of the matrix.

Algorithms such as projective gradient and multiplicative iteration [21] are used to solve the NMF problems, these algorithms minimize the objective function starting from two non-negative matrices and iterate continuously, and the process decreases. Although the minimization problem of Equation (1) is separately convex in A and S, it is not simultaneously convex in both matrices. The widely used multiplicative algorithm presented in [10] is simple to implement and performs well, and can be generated from the traditional gradient descent algorithm. However, Equation (4) has a non-unique solution due to its non-convex for A and S. For example, if the solutions to Equation (4) are A and S, then there exist invertible matrices B such that $A_{1}=AB$ and $S_{1}=B^{-1}S$ are also solutions of the equation. Therefore, the solutions are not unique, which is the biggest disadvantage of the NMF-based algorithm. The two most commonly used methods to solve this problem are to assign appropriate initial values and add constraints [21].

In this research, the traditional NMF method or the unconstrained NMF is applied, where no abundance and end-element constraints are imposed. The purpose of using the unconstrained NMF method is to consider a scenario when the smoothness of the end-element spectra is unknown. Therefore, an approximation of the smoothness for the end-element spectra can provide guidance or flag to drive the following piecewise smoothness on the end-element spectra. It is noticed that in the proposed method, the solution’s uniqueness cannot be guaranteed. However, if the initial spectra are assumed appropriately, the following solution can be improved significantly and is acceptable for many applications.

## 3. Sparse and Smooth Constrained NMF Method

### 3.1. The Sparseness of the Abundance

In most cases, any end member does not contribute to all pixels in the scene. Thus, the abundance matrix is sparse. The $L_{0}$ norm is applied to the objective function to promote the sparsity of the abundance matrix, but it suffers from the NP-hard problem. $L_{1}$ or $L_{P}(0.5\leq p<1)$ constraints are proposed by related researchers. Although $L_{1}$ regularization is widely used, the constraint of sum 1 is often not satisfied. In the proposed method, the $L_{1}$ norm is applied since no significant difference has been found between the choices of $L_{1}$ and $L_{1/2}$ norm in our simulations. It may indicate that the selection of a proper norm in spectral unmixing is a dataset-dependent problem.

Reweighted sparse regularization [14] is applied to the NMF model in this paper to promote the sparsity of the abundance matrix. Specifically, the performance of the $L_{1}$ minimization framework is improved by weighting the $L_{1}$ parameterization and iteratively updating the weights. The weighted $L_{1}$ minimization problem can be expressed as follows:

(5) $$\min_{s}{\left\|\omega .*s\right\|}_{1}\;s.t.\;y=As$$

where ∗ represents the element-wise multiplication. $\omega \in R^{K\times 1}$ is the weight vector. By proving that the weighted $L_{1}$ regularizer can obtain a sparser solution than the $L_{1}$ regularizer with a suitable vector of weights. However, how to set the weights is a crucial problem. Candes et al. [22] proposed an iterative reweighting algorithm to solve a series of weighted $L_{1}$ minimization problems, in which the weights for the next iteration are calculated based on the current abundance matrix, i.e.,

(6) $$W_{i,j}^{\left(k+1\right)}=1./(\left|S_{i,j}^{\left(k\right)}\right|+\mathrm{eps})$$

where $S_{i,j}^{\left(k\right)}$ denotes the abundance matrix of the kth iteration, and eps is a positive constant, ./ represents the element-wise division.

### 3.2. The Smoothness of the Abundance

The rows of the abundance matrix are smooth. This is due to the similar fractional abundances in adjacent pixels of the same end member. However, discontinuities may occur owing to the abrupt changing of end-member abundance in spatial space. Therefore, the spectral data are piecewise smooth in spatial domains. In this paper, Total Variation (TV) regularization is used to facilitate the piecewise smoothing property, and the process can be regarded as an abundance map denoising process. TV regularization was first proposed by Rudin et al. [23] for solving the grayscale map denoising problem, and for a grayscale image y of size m×n, the TV parametrization is defined as

(7) $${\left\|y\right\|}_{TV}=\sum_{i=1}^{m-1}\sum_{j=1}^{n-1}\left\{\left|y_{i,j}-y_{i+1,j}\right|+\left|y_{i,j}-y_{i,j+1}\right|\right\}+\sum_{i=1}^{m-1}\left|y_{i,n}-y_{i+1,n}\right|+\sum_{j=1}^{n-1}\left|y_{m,j}-y_{m,j+1}\right|$$

For an HSI, every band of the HSI can be treated as a grayscale image, so the hyperspectral total variance regularization is defined as [14]

(8) $${\left\|S\right\|}_{HTV}=\sum_{j=1}^{K}{\left\|FS^{j}\right\|}_{TV}$$

where K is the number of end members in the image and $S^{j}$ represents the row vector form of the jth band of the HSI. If the number of pixels in the HSI is N, F represents the operator that reshapes the row vector (with a total of N pixels) as a matrix of m×n, i.e., N=m×n.

### 3.3. The Smoothness of the End member

HSI has high spectral resolution, and the adjacent bands have similar spectral reflection values, so the end-member spectra of HSI have a certain degree of smoothness. However, the removal of the noise band may cause abrupt changes in the reflection values of adjacent bands, so the end-member spectral curve has piecewise smoothness. First of all, this paper obtains the estimated value of the end-member signatures Aest through an unconstrained NMF, which is used as a priori information to determine the degree of smoothing of the end-member matrix A. That is, different smoothing levels are assigned according to the differences in the reflectance values of adjacent bands of Aest, where the smoothing levels are described by the weight matrix Q, which is defined as

(9) $$Q_{i,j}=e^{-\frac{{\left(Aest_{i,j}-Aest_{i+1,j}\right)}^{2}}{\sigma }}$$

where $Aest_{i,j}$ is the ith row and jth column element of the end-member matrix estimated using the unconstrained NMF algorithm, i.e., the spectral reflection value corresponding to the ith band in the jth end-member signature. σ is the parameter that controls the degree of smoothing, and obviously, the closer $Aest_{i,j}$ is to $Aest_{i+1,j}$, the larger the weight $Q_{i,j}$ is.

**Figure 1:**
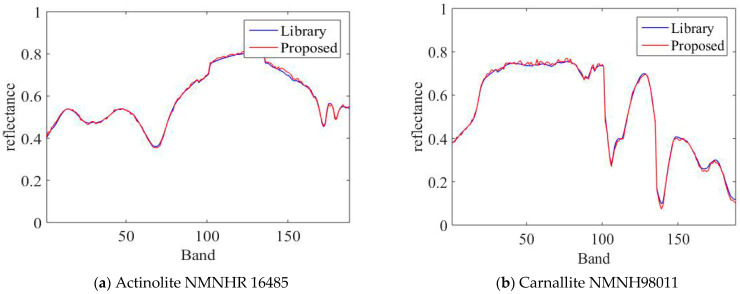
Comparison of the two end-members spectra obtained using NMF estimation in the simulated data with the real end-members spectra.

Figure 1 shows the comparison of the end-element signatures extracted by the unconstrained NMF method with the reference spectra. From the figure, it can be seen that the estimated end-member signatures are very similar to the reference signatures and their trends are basically the same. Therefore, the estimated signatures can be used as a priori information to measure the smoothness of the end-member spectra.

The end-member spectral piecewise smoothness regularization is defined as

(10) $$\mathrm{minimize}\;J_{1}(A)=\sum_{j=1}^{K}\sum_{i=1}^{L-1}Q_{i,j}{\left(A_{i,j}-A_{i+1,j}\right)}^{2}$$

where K is the number of end member, L is the number of spectral bands, and $A_{i,j}$ is the spectral reflection value of the ith band corresponding to the jth end-member signature to be estimated.

### 3.4. Smoothing and Sparse Constraints-NMF(SSC-NMF) HU Model

According to the above discussion about the characteristics of HSI, the SSC-NMF model proposed in this paper is obtained by integrating the piecewise smoothness constraint and sparsity constraint into the NMF model. The SSC-NMF model is expressed as

(11) $$\begin{array}{l} \mathrm{minimize}\;J(A,S)=\;\frac{1}{2}{\left\|Y-AS\right\|}_{F}^{2}+\lambda {\left\|W.*S\right\|}_{1}+\tau {\left\|S\right\|}_{HTV}+\beta J_{1}(A)\\ \mathrm{subject}\;\mathrm{to}\;A\geq 0,S\geq 0,1_{K}^{T}S=1_{N}^{T} \end{array}$$

where the first term is the standard fidelity term, parameter λ controls the sparsity of the abundance matrix, and parameter τ controls the piecewise smoothness of the abundance matrix. The last term promotes the piecewise smoothness of the end-member spectra, and parameter β controls the piecewise smoothness of the end-member matrix.

### 3.5. Model Optimization

This paper introduces an auxiliary variable L to make better use of the multiplicative iteration rule. The objective function is then transformed into

(12) $$\begin{array}{l} \mathrm{minimize}\;J(A,S,L)=\;\frac{1}{2}{\left\|Y-AS\right\|}_{F}^{2}+\lambda {\left\|W.*S\right\|}_{1}+\tau {\left\|L\right\|}_{HTV}+\beta J_{1}(A)\\ \mathrm{subject}\;\mathrm{to}\;A\geq 0,S\geq 0,1_{K}^{T}S=1_{N}^{T},L=S \end{array}$$

If we take S as a noisy version of the auxiliary variable L, then the constraint L=S can be integrated into the objective function thus we obtain the following problem

(13) $$\begin{array}{l} \mathrm{minimize}\;J(A,S,L)=\;\frac{1}{2}{\left\|Y-AS\right\|}_{F}^{2}+\lambda {\left\|W.*S\right\|}_{1}+\frac{\mu }{2}{\left\|L-S\right\|}_{F}^{2}+\tau {\left\|L\right\|}_{HTV}+\beta J_{1}(A)\\ \mathrm{subject}\;\mathrm{to}\;A\geq 0,S\geq 0,1_{K}^{T}S=1_{N}^{T} \end{array}$$

where parameter μ is used as the penalty parameter to control the similarity of L and S. For the optimization problem of Equation (13), it can be decomposed into three sub-problems to optimize A,S,L respectively.

(14) $$\begin{array}{l} A=\underset{A}{\arg\min}J(A,S,L)\\ S=\underset{S}{\arg\min}J(A,S,L)\\ L=\underset{L}{\arg\min}J(A,S,L) \end{array}$$

The proposed method consists of three steps: (1) an end-member estimation step, (2) an abundance estimation step, and (3) an abundance denoising step. In each step, the value of one variable is updated according to the current values of the other variables so that the value of the objective function iteratively decreases. The following is a more detailed description.

### 3.6. Update Rules

#### 3.6.1. End-Members Estimation

The optimization problem of A can be formulated as

(15) $$\mathrm{minimize}\;J(A)=\frac{1}{2}{\left\|Y-AS\right\|}_{F}^{2}+\beta J_{1}(A)\;\;s.t.\;A\geq 0$$

In Equation (15), the constrained problem is transformed into an unconstrained problem using the Lagrange multiplier method

(16) $$\mathrm{minimize}\;J(A)=\;\frac{1}{2}{\left\|Y-AS\right\|}_{F}^{2}+\beta J_{1}(A)+\mathrm{Tr}(\psi A^{T})$$

where $\psi \in R^{B\times K}$ is the Lagrange multiplier in matrix format. Based on the Karush–Kuhn–Tucker (KKT) conditions, we obtain the following linear equations:

(17) $$\frac{\partial J(A)}{\partial A}=ASS^{T}-YS^{T}+\beta \frac{\partial J_{1}(A)}{\partial A}+\psi =0$$

(18) A.∗ψ=0

If both sides of Equation (17) are simultaneously multiplied by matrix A, we can obtain the following equation:

(19) $$A.*(ASS^{T})-A.*(YS^{T})+A.*(\beta \frac{\partial J_{1}(A)}{\partial A})+A.*\psi =0$$

We obtain the following update rule for A by combining Equation (18) with Equation (18)

(20) $$A\leftarrow A.*(YS^{T}-\beta \frac{\partial J_{1}(A)}{\partial A})./ASS^{T}$$

where the derivative of $J_{1}(A)$ with respect to each element in A is

(21) $$\frac{\partial J_{1}(A)}{\partial A_{i,j}}=2(A_{i,j}-A_{i+1,j})*Q_{i,j}$$

The third condition is satisfied in the manuscript by ensuring the non-negativity of A during initialization. According to Equations (4) and (5), the update rules for SSC-NMF can be formulated as follows:

$$A\leftarrow A-\mu_{A}(ASS^{T}-YS^{T}+\beta \frac{\partial J_{1}(A)}{\partial A})$$

$$S\leftarrow S-\mu_{S}*(A^{T}AS-A^{T}Y+\lambda W+\mu (S-L))$$

where $\mu_{A}$ and $\mu_{S}$ are the step sizes. They are set as $\mu_{A}=A./ASS$ and $\mu_{S}=S./AAS$ to meet the non-negative constraints. Thus, the update rules can be obtained as follows:

$$A\leftarrow A.*(YS^{T}-\beta \frac{\partial J_{1}(A)}{\partial A})./ASS^{T}$$

$$S\leftarrow S.*(A_{}^{T}Y+\mu L)./(A_{}^{T}A_{}S+\lambda W+\mu S)$$

The initialization of A and S should be non-negative to ensure their non-negativity during the iteration under the rules presented by Equations (20) and (24). The cost function Equation (15) is non-increasing under the update rules, and it will be convergent to a stationary point [24].

#### 3.6.2. Abundance Estimation

Similarly, the optimization problem of S can be formulated as

(22) $$\mathrm{minimize}\;J(S)=\frac{1}{2}{\left\|Y-AS\right\|}_{F}^{2}+\lambda {\left\|W.*S\right\|}_{1}+\frac{\mu }{2}{\left\|L-S\right\|}_{F}^{2}\;s.t.\;S\geq 0$$

The augmentation matrix Y and A in each iteration is given by the following equation to satisfy the abundance sum-to-one constraint (ASC).

(23) $$\bar{Y}=\left[\begin{array}{c} Y\\ \delta 1 \end{array}\right],\;\;\bar{A}=\left[\begin{array}{c} A\\ \delta 1 \end{array}\right]$$

where δ controls the effect of ASC constraint, 1 denotes the all-1 row vector, Equation (22) is an inequality constrained optimization problem, and the iterative formula for the abundance matrix S is obtained by basing the KKT condition on

(24) $$S\leftarrow S.*({\bar{A}}_{}^{T}\bar{Y}+\mu L)./({\bar{A}}_{}^{T}{\bar{A}}_{}S+\lambda W+\mu S)$$

For the validity of the update rule shown in Equation (23), please refer to [14].

#### 3.6.3. Abundance Denoising

The optimization problem for L can be formulated as

(25) $$\mathrm{minimize}\;L=\frac{\mu }{2}{\left\|L-S\right\|}_{F}^{2}+\tau {\left\|L\right\|}_{HTV}$$

Equation (25) is equated to solve the following problem

(26) $$\mathrm{minimize}\;J(L)=\sum_{j=1}^{K}\left(\frac{\mu }{2}{\left\|FL_{}^{j}-FS_{}^{j}\right\|}_{F}^{2}+\tau {\left\|FL_{}^{j}\right\|}_{TV}\right)$$

Further translating Equation (26) into K standard TV smoothing problems

(27) $${\hat{L}}_{}^{j}=\underset{L_{}^{j}}{\min}\frac{\mu }{2}{\left\|FL_{}^{j}-FS_{}^{j}\right\|}_{F}^{2}+\tau {\left\|FL_{}^{j}\right\|}_{TV},j=1,\cdots ,K$$

In this paper, the fast gradient projection algorithm (FGP) [25] is used to solve Equation (27), such as the following Algorithm 1.


**Algorithm 1 Smoothing and Sparse Constraints NMF for HU**
1. **Input:** The observed mixture data $Y\in R^{K\times N}$, the number of end-members K, the maximum number of iterations c, the parameters λ,β,μ,τ,σ. 2. **Output:** End-member signature matrix A and abundance matrix S. 3. **Initialize** A,S,L, and weighted matrix W. 4. **Repeat** until convergence: 5. Update the weight matrix W with Equation (6); 6. Using Equation (19) to update A; 7. Obtain the augmentation matrix of A and S respectively using Equation (23) 8. Update S by Equation (24); 9. Update L with Equation (27).

## 4. Experimental Results and Discussion

### 4.1. Simulated Data Experiments

**Figure 2:**
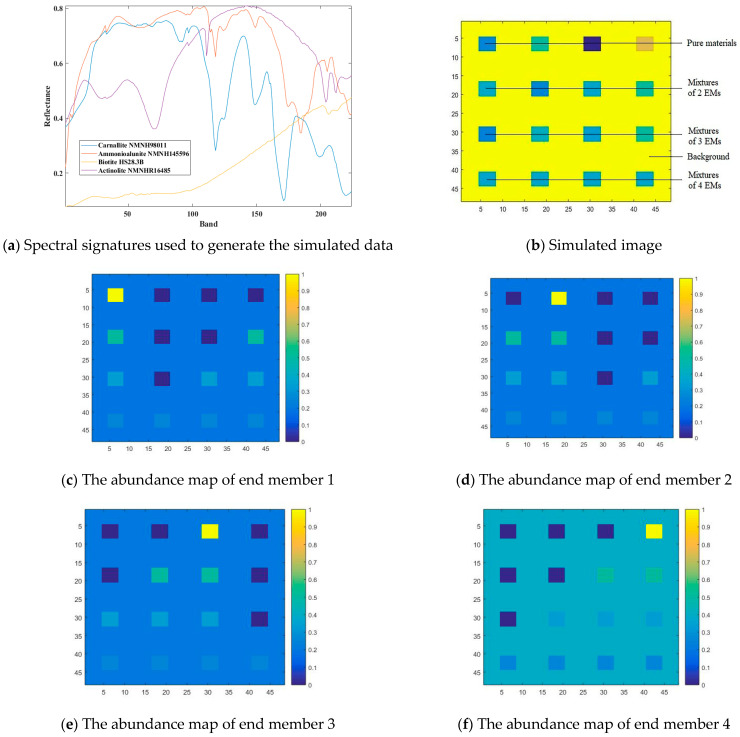
Spectral signatures used to generate the simulated data and abundance map of end member, 1–4.

In the simulated data experiment, four end-member spectra from the United States Geological Survey (USGS) digital spectral library are selected to apply to the simulation data experiment. They are linearly mixed in a certain proportion to obtain simulation data. In this research, the mixture proportions are listed in Table 1:

As shown in Figure 2a, each spectral curve includes the spectral reflectance corresponding to 224 spectral bands in the wavelength range of 0.4–2.5 μm. By removing the noise and water absorption bands, only the remaining 188 low-noise bands are selected to synthesize the simulated data. After linear mixing, 48∗48 pixels are generated, and each pixel has 188 bands. The simulated image is shown in Figure 2b; the pure pixel area is displayed in the first row and areas with different levels of mixing are displayed in 2–4 lines. In addition, the background pixels are also mixed with four types of end members in different proportions, where different colors indicate different degrees of mixing. In Figure 2c–f, it is found that the more yellow color means the higher proportion of a certain end member in the region.

#### 4.1.1. Parameter Selection

The selection of parameter λ is related to the sparsity of the dataset. This paper refers to a method proposed by Qian [12] for estimating the sparse regularization parameter, which is defined as

(28) $$\lambda_{e}=\frac{1}{\sqrt{L}}\sum_{l=1}^{L}\frac{\sqrt{N}-{\left\|Y_{l}\right\|}_{1}/{\left\|Y_{l}\right\|}_{2}}{\sqrt{N}-1}$$

where L denotes the number of bands, N denotes the number of pixels, and $Y_{l}$ denotes the vector corresponding to the lth band.

Parameter τ is chosen to be related to the abundance smoothing of the dataset. Therefore, the similarity of the neighboring pixels is evaluated based on the similarity of their spectral values, and then the smoothness of the abundance map is estimated, which is defined as

(29) $$\tau_{e}=\frac{1}{N}\sum_{i=1}^{N-1}{\left\|x_{i}-x_{i+1}\right\|}_{}^{2}$$

where N denotes the number of pixels, and $x_{i}$ denotes the spectral vector corresponding to the ith pixel.

**Figure 3:**
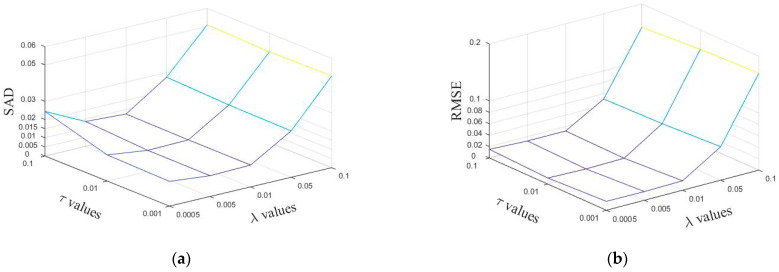
Performance of SSC-NMF with respect to parameters λ,τ in terms of (**a**) SAD (Spectral Angle Distance) and (**b**) RMSE (Root Mean Square Error).

When the other parameters are fixed, we discuss the influence of the sparsity parameter λ and the TV regularization parameter τ on the experimental results of the simulation data based on Equations (28) and (29). The results are shown in Figure 3. From this figure, it can be seen that better results can be obtained when λ and τ take smaller values, so choosing the right parameters will have a positive impact.

From Figure 3, it can be obtained that the optimal parameter of λ in the proposed method is less than $\lambda_{e}$, and the optimal parameter is within [$\lambda_{e}$/10, $\lambda_{e}$]. The optimal parameter of τ is optimized within the next order of magnitude of $\tau_{e}$/10 to find the optimal value. Considering both RMSE (Root Mean Square Error) and SAD (Spectral Angle Distance), the λ parameter of the proposed method in this paper is set to 0.005 and τ is set to 0.01 in the simulated data experiments.

**Figure 4:**
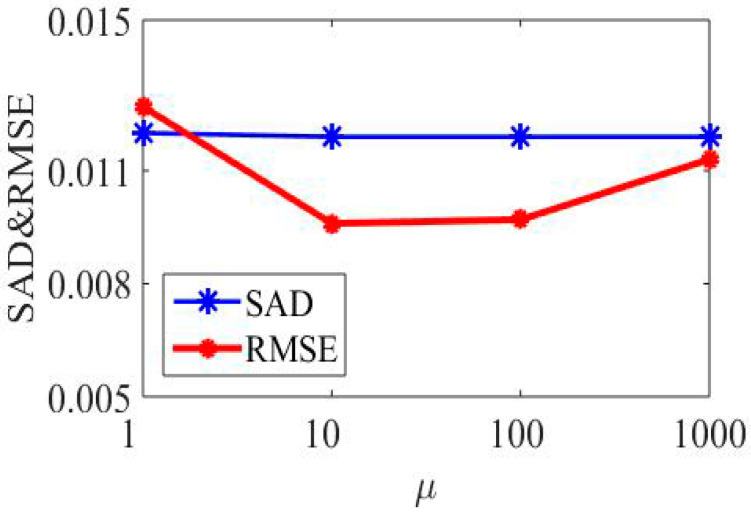
Performance of SSC-NMF with respect to parameter μ in terms of SAD and RMSE.

Secondly, parameter μ is the penalty parameter for violating the linear constraint L=S. The larger it is, the closer Equations (12) and (13) are. In this paper, the value of μ varies between 1, 10, 100, and 1000, and the results are shown in Figure 4. RMSE and SAD are two metrics that show the average distance between the predicted values from the model and the actual values in the dataset. The lower the RMSE and SAD, the better an algorithm is able to unmix the data. It can be seen from Figure 4 that RMSE first decreases and then increases when the value of μ becomes larger, and the SAD value basically remains the same in the simulated dataset. However, for each dataset, the choice of μ is different, and the best value is detected by searching for parameters within an order of magnitude above 100.

**Figure 5:**
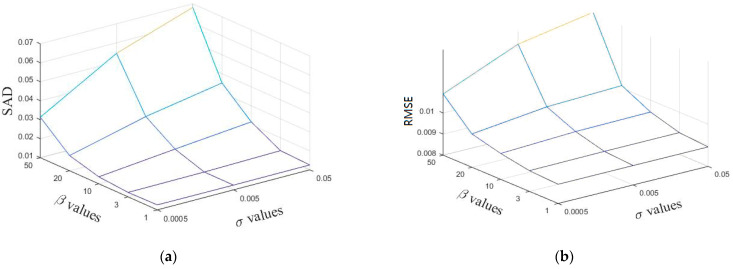
Performance of SSC-NMF with respect to parameters β,σ in terms of (**a**) SAD and (**b**) RMSE.

Finally, the effect of parameter β,σ on the experimental results is discussed, and the results are shown in Figure 5. It can be seen from the figure that SAD and RMSE decrease continuously as β decreases, and finally it reaches a stable state. The value of β is generally taken as around 10 for different datasets due to the different spectral resolutions. For parameter σ, the searching parameter is usually performed by an order of magnitude above and below 0.005. The parameters of other reference methods in this paper are determined according to the analysis in the corresponding references.

#### 4.1.2. Robustness Analysis

To study the robustness of the proposed method to noise, this paper verifies the performance of the proposed method under different noise levels by adding noise to the simulated data. By setting three different signal-to-noise ratios, three different levels of noise data are obtained, which are 15, 25, and 35 dB, respectively, where the signal-to-noise ratio is defined as

(30) $$\mathrm{SNR}=10\log_{10}\frac{E[x^{T}x]}{E[n^{T}n]}$$

where x,n is the observed value of the image and the noise, respectively, and E[⋅] denotes the expectation operator.

The SAD and RMSE obtained by the four methods with Gaussian noise at different signal-to-noise ratios are listed in Table 2 and Table 3, respectively. The benchmarked methods include TV-RSNMF [14], *L*_1/2_NMF, and VCA-FCLS, where VCA-FCLS is a supervised-based unmixing method that first uses VCA for end element extraction and then uses the FCLS method for abundance estimation. *L*_1/2_NMF and TV-RSNMF methods are unsupervised-based unmixing methods, where the *L*_1/2_NMF method enhances the sparsity of the unmixing by imposing sparsity constraints in the NMF objective function, and the TV-RSNMF method considers both the sparsity of abundance and segmental smoothing. When the signal-to-noise ratio is increased to a higher level, such as 35 dB, the majority of the methods, including SSC-NMF, TV-RSNMF, and *L*_1/2_NMF, achieved similar results. In this case, the proposed method is not the best one, but the difference is insignificant. This reveals that the proposed method is more suitable for those harder applications where the signal-to-noise ratio is lower than 35 dB.

**Figure 6:**
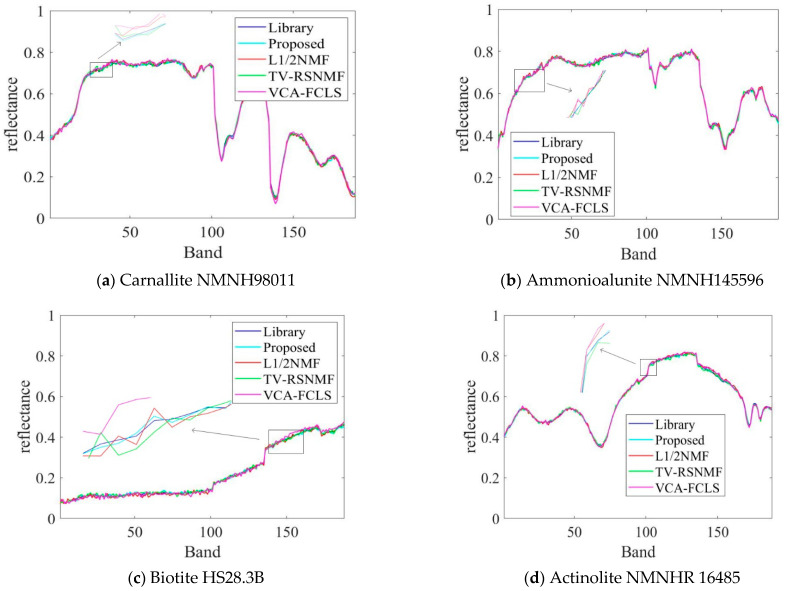
Comparison of the end-member signatures extracted by the SSC-NMF method with the reference spectra.

**Figure 7:**
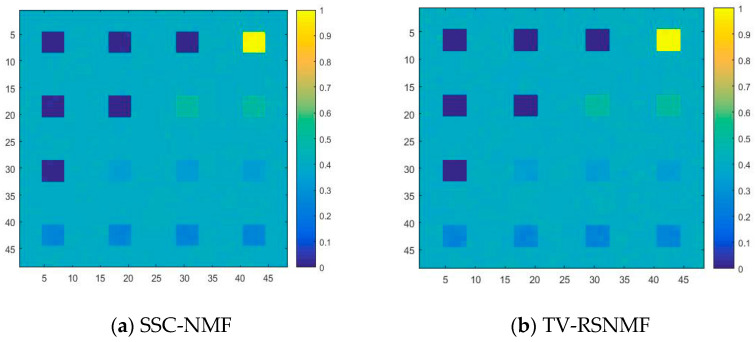

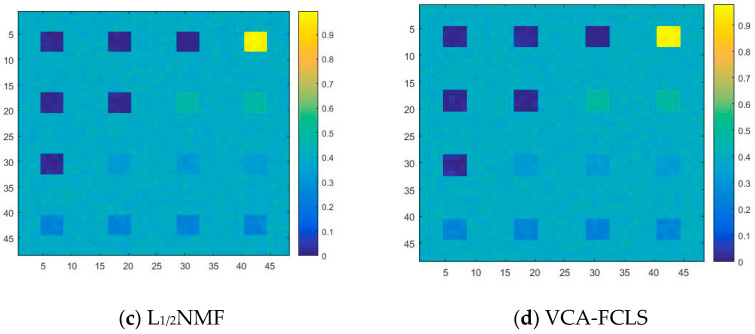
Comparison of abundance maps of the fourth end member estimated by different methods.

For the convenience of comparison, the optimal results in this paper are labeled in bold. It can be seen from Table 2 that the SAD and RMSE values obtained by the proposed method are lower compared to TV-RSNMF [14], *L*_1/2_NMF [12], and VCA-FCLS [6], indicating that the algorithm is better at extracting the end-member signatures and inversion abundance. When SNR = 15 dB, the *L*_1/2_NMF method obtains the highest RMSE value, mainly due to the fact that the *L*_1/2_NMF method cannot handle the low SNR case. The VCA-FCLS method tends to treat noise as an end member when the noise level is high because this method treats monomorphic vertices as an end member, which largely limits the performance of HU. However, the disadvantage of VCA-FCLS is not obvious when the signal-to-noise ratio is low. In addition, the TV-RSNMF method achieves better performance than the *L*_1/2_NMF method due to the *L*_1/2_NMF method only considering the sparsity constraint of abundance and ignoring the smoothness constraint of abundance, thus indicating the superiority of TV-RSNMF. Further, the end-member piecewise smoothness constraint with TV-RSNMF further improves the accuracy of HU, illustrating the effectiveness of the method proposed in this paper. Figure 6 shows a comparison of the end-member signatures extracted by the different methods with the reference signature. It is seen in Figure 6 that there is a significant deviation between the results obtained by the VCA-FCLS method and the reference value. On the other hand, the end-member signatures extracted by TV-RSNMF and SSC-NMF are in good agreement with the reference signature. Overall, the proposed method achieved a smaller difference with the reference spectrum compared with other benchmarked methods. Compared with different methods for estimating abundance maps of the fourth end member, the most accurate abundance estimation is obtained from the method of SSC-NMF in Figure 7.

### 4.2. Real Data Experiments

**Figure 8:**
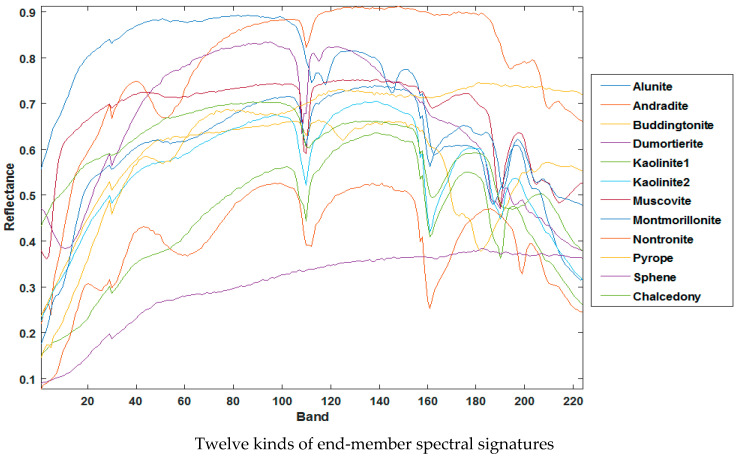
Twelve kinds of end-member signature from Cuprite hyperspectral dat. (Courtesy of NASA AVIRIS dataset at http://aviris.jpl.nasa.gov/ (accessed on 10 December 2021)).

Cuprite dataset: The Cuprite real dataset is used for experiments, which is the benchmark dataset in the HU study. The original cuprite dataset contains 224 bands that cover the wavelength range of 0.37–2.48 μm. In the experiment, noisy bands and water-absorption bands were removed before the unmixing, leaving 188 bands in total. We choose 250 × 190 regions for the experiment, and there is no accurate answer for the most terminal end-member number due to the strong spectral variability of the AVIRIS Cuprite scene and the presence of a large amount of mineral alteration in the scene. In this paper, the number of end members is set to 12 by referring to other articles [14], where the end-member signatures are shown in Figure 8. It is worth noting that only the end-member signatures are known as a priori for this dataset; the abundance map is unknown.

Considering that the spectra in the USGS spectral library are obtained under ideal conditions, and the spectra in real scenes are affected by atmospheric interference and other environmental factors, there are inevitably some differences between the end-member signatures extracted by SSC-NMF and the reference spectra. Table 4 presents the SAD values achieved by the different HU methods, including TV-RSNMF [14], *L*_1/2_NMF [12], VCA-FCLS [6], and the recently developed ULTRA-V [19]. From the table, it can be seen that most of the end members obtained by SSC-NMF have optimal or sub-optimal SAD. The average SAD of the proposed method is significantly lower than those of the TV-RSNMF, *L*_1/2_NMF [12], VCA-FCLS [6], and is slightly lower than the ULTRA-V. This can indicate the superiority of this algorithm.

**Figure 9:**
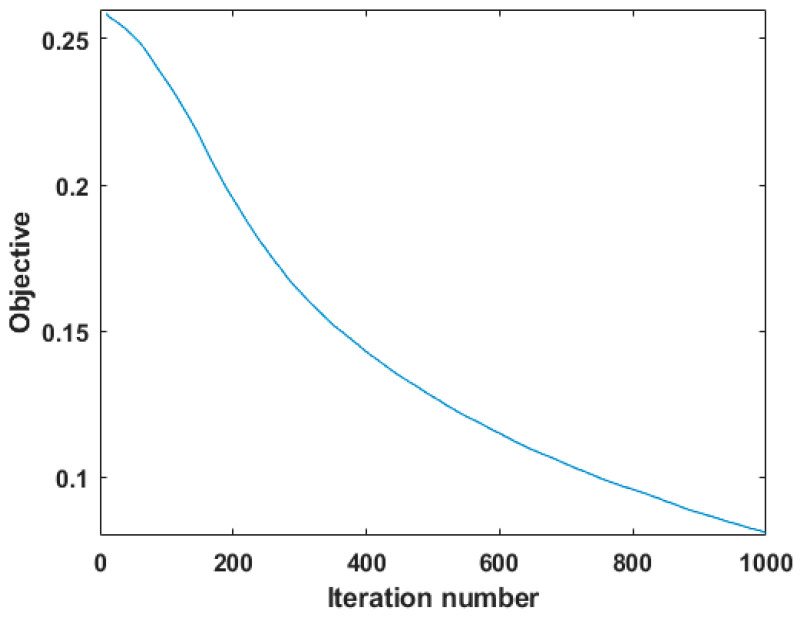
Illustration of convergence of the optimization.

Figure 9 shows the convergence curve of the algorithm, where the *x*-axis stands for the iteration number and the *y*-axis denotes the objective value discussed in Equation (12). As seen in Figure 9, the objective value decreases monotonically with the algorithm iteration. Therefore, it can be expected that the unmixing results will approach true values step by step.

## 5. Discussion and Conclusions

SSC-NMF, a new HU model, is proposed in this paper to improve the problems of traditional NMF-based models in unmixing applications. This method has the advantages of fully considering the characteristics of HSI, integrating the end-member piecewise smoothness, abundance smoothness and sparsity constraints into the NMF model to further limit the solution space. Especially in the case of a low signal-to-noise ratio, the method proposed in this paper has better robustness, as proven by the simulation experiments. The SSC-NMF model is solved by multiplication and iteration rules. The results from experiments of the simulated data and Cuprite real data show that this method has improved performance compared with other methods, which demonstrates that the integration of abundance and end-member features in SSC-NMF is effective. However, the method proposed in this paper still has some shortcomings. For example, the computing complexity of the proposed methods is similar to the TV-RSNMF method and *L*_1/2_NMF method, but it is relatively higher than the VCA-FCLS method. Moreover, selecting parameters is an important step in this method, which requires a sufficient training dataset and becomes a disadvantage for real-time applications. In this research, the parameters are optimized within a certain range. Therefore, the next focus will be on how to realize the adaptive problem of smoothing parameters. Moreover, only the class labels for each pixel are given in the ground truth associated with the Cuprite dataset, leaving us without the true end-member spectral data and abundance data. In future research, we will test the proposed method on other datasets where the abundance and end-member information are included.

## Figures and Tables

**Table 1:** Mixture proportions of the simulated data.

Mixture Proportion (%)	End Member 1 (Carnallite)	End Member 2 (Ammonioalunite)	End Member 3 (Biotite)	End Member 4 (Actinolite)
Case 1	20	20	20	40
Case 2	33	33	33	0
Case 3	0	33	33	33
Case 4	25	25	25	25

**Table 2:** SAD values of different methods with the simulated data.

SNR/dB	SSC-NMF	TV-RSNMF	L_1/2_NMF	VCA-FCLS
15	0.0389	0.0397	0.0440	0.0564
25	0.0116	0.0125	0.0149	0.0167
35	0.0046	0.0047	0.0046	0.0049

**Table 3:** RMSE values of different methods with the simulated data.

SNR/dB	SSC-NMF	TV-RSNMF	L_1/2_NMF	VCA-FCLS
15	0.0378	0.0418	0.0492	0.0473
25	0.0092	0.0104	0.0158	0.0171
35	0.0057	0.0058	0.0059	0.0074

**Table 4:** SAD values of different methods with the real Cuprite dataset.

Method	SSC-NMF	TV-RSNMF	L_1/2_NMF	VCA-FCLS	ULTRA-V
Alunite	0.1049	0.1064	0.0921	0.0859	0.0842
Andradite	0.0872	0.0878	0.0652	0.0582	0.0511
Buddingtonite	0.0972	0.0964	0.0648	0.0724	0.0571
Dumortierite	0.1086	0.1112	0.0972	0.0978	0.0991
Kaolinite1	0.1316	0.1316	0.1268	0.1222	0.1778
Kaolinite2	0.0450	0.0449	0.0440	0.0458	0.0481
Muscovite	0.1278	0.1279	1.1667	1.1522	0.8819
Montmorillonite	0.0696	0.0698	0.0720	0.0717	0.0919
Nontronite	0.0902	0.0904	0.1173	0.1070	0.1379
Pyrope	0.0879	0.0881	0.1897	0.1783	0.1421
Sphene	1.0130	1.0130	0.0826	0.0876	0.0897
Chalcedony	0.0695	0.0761	0.1919	0.1675	0.1771
Mean	0.1694	0.1703	0.1925	0.1872	0.1698

## Data Availability

A publicly archived dataset Cuprite can be downloaded from https:/www.ehu.eus/ccwintco/index.php/Hyperspectral_Remote_Sensing_Scenes (accessed on 20 December 2021).
